# Comparative study on the effects of substrate stiffness on cell morphology and focal adhesion expression between hMSCs and AFSCs

**DOI:** 10.1186/1753-6561-6-S4-O30

**Published:** 2012-07-09

**Authors:** B Lowry, R McCoy, F O'Brien

**Affiliations:** 1 Royal College of Surgeons in Ireland

## Introduction

Stiffness plays an important role in cell differentiation. Our project set out to compare the effects of substrate stiffness on cell morphology and focal adhesion expression between hMSCs and AFSCs.

## Methods

We developed a novel technique for fabricating thin collagen-GAG films, which were further stiffened via DHT cross-linking techniques and/or EDAC cross-linking techniques. We cultured both cell types either on the fabricated collagen-GAG films or on glass microscope-slide cover-slips that had been coated in collagen-GAG slurry.

## Results

Staining and imaging showed numerous clear focal adhesions had been formed for both cell types and the general morphology of the cells represented that of healthy cells with a flatly spread appearance when cultured on the glass cover-slips. On the collagen films, the cells showed poor focal adhesion formation and very diffuse staining, but no difference was observed as a function of cross-linking technique. Furthermore, the low stiffness also allowed the cells to contract giving them a narrow, elongated morphology.

## Conclusions

In conclusion, from the limited number of cells observed in this study no qualitative differences were noted between AFSCs and MSCs in terms of focal adhesion expression or cell morphology on substrates of varying stiffness. However, focal adhesion expression levels were markedly reduced for both cell types on the less stiff collagen-GAG films.

